# Unbiased insights into the multiplicity of the CYP46A1 brain effects in 5XFAD mice treated with low dose-efavirenz

**DOI:** 10.1016/j.jlr.2024.100555

**Published:** 2024-05-06

**Authors:** Natalia Mast, Makaya Butts, Irina A. Pikuleva

**Affiliations:** Department of Ophthalmology and Visual Science, Case Western Reserve University, Cleveland, OH, USA

**Keywords:** brain lipids, cholesterol metabolism, oxysterols, proteomics, glycerophospholipids

## Abstract

Cytochrome P450 46A1 (CYP46A1) is the CNS-specific cholesterol 24-hydroxylase that controls cholesterol elimination and turnover in the brain. In mouse models, pharmacologic CYP46A1 activation with low-dose efavirenz or by gene therapy mitigates the manifestations of various brain disorders, neurologic, and nonneurologic, by affecting numerous, apparently unlinked biological processes. Accordingly, CYP46A1 is emerging as a promising therapeutic target; however, the mechanisms underlying the multiplicity of the brain CYP46A1 activity effects are currently not understood. We proposed the chain reaction hypothesis, according to which CYP46A1 is important for the three primary (unifying) processes in the brain (sterol flux through the plasma membranes, acetyl-CoA, and isoprenoid production), which in turn affect a variety of secondary processes. We already identified several processes secondary to changes in sterol flux and herein undertook a multiomics approach to compare the brain proteome, acetylproteome, and metabolome of 5XFAD mice (an Alzheimer’s disease model), control and treated with low-dose efavirenz. We found that the latter had increased production of phospholipids from the corresponding lysophospholipids and a globally increased protein acetylation (including histone acetylation). Apparently, these effects were secondary to increased acetyl-CoA production. Signaling of small GTPases due to their altered abundance or abundance of their regulators could be affected as well, potentially via isoprenoid biosynthesis. In addition, the omics data related differentially abundant molecules to other biological processes either reported previously or new. Thus, we obtained unbiased mechanistic insights and identified potential players mediating the multiplicity of the CYP46A1 brain effects and further detailed our chain reaction hypothesis.

CYP46A1 (cytochrome P450 46A1) is a CNS-specific enzyme, expressed in the neurons of the brain and retina (1, 2, 3, 4). In the brain, CYP46A1 is the major cholesterol hydroxylase, catalyzing the conversion of cholesterol into 24-hydroxycholesterol (24HC) (1). Cholesterol cannot cross the blood-brain barrier, therefore, the formation of 24HC represents a mechanism for cholesterol removal from the brain, which utilizes 75%–85% and 40%–50% of the excess brain cholesterol in humans and mice, respectively (5, 6). Once it is formed, 24HC is fluxed from the brain into the systemic circulation and is delivered to the liver for subsequent metabolism (7). Cholesterol maintenance in the brain is independent from that in the whole body and tightly couples the brain’s 24HC production with in situ cholesterol biosynthesis, both of which control the brain cholesterol turnover (6, 8, 9).

CYP46A1 activity could be modulated by gene therapy or pharmacologically (reviewed in (10, 11, 12, 13)), and the therapeutic potential of CYP46A1 has been already evaluated in clinical trials (14, 15, 16) as well as animal models (17, 18, 19, 20, 21, 22, 23, 24, 25, 26, 27, 28). Studies on animal models revealed that CYP46A1 activity modulation mitigated the manifestations of both neurodegenerative and nonneurodegenerative diseases with both activation and inhibition of CYP46A1 being of a therapeutic value. Specifically, increases in CYP46A1 activity were beneficial in mouse models of Alzheimer's disease (AD), Huntington's, Niemann-Pick type C, and Machado-Joseph (spinocerebellar ataxia type 3) diseases, amyotrophic lateral sclerosis, glioblastoma, depression, epileptic seizures, and prion infection (17, 18, 19, 20, 21, 22, 23, 24, 25, 26, 29, 30, 31), whereas CYP46A1 inhibition reduced seizures in mice with Dravet syndrome and animals infected with the Theiler's encephalomyelitis virus (27, 28). Moreover, the positive CYP46A1 inhibition effect on specific seizure types was confirmed in clinical trials (14, 15, 32).

Remarkably, processes affected by CYP46A1 activity changes extent beyond cholesterol-related and encompass those, which are common in neurodegenerative diseases (e.g., accumulation of misfolded proteins, memory and motor function, gene expression, protein phosphorylation, autophagy, and lysosomal processing) and those, which were altered only under certain pathologic conditions (e.g., apoptosis, stress of the endoplasmic reticulum, transcriptional regulation via liver X receptors, expression of synaptic proteins, stimulation of dopaminergic neurogenesis, function of lipid rafts, and other, (reviewed in Refs. (8, 11, 33)). Thus, various brain diseases and apparently unlinked biological processes could be affected by CYP46A1 activity modulation, raising a question of how one enzyme, CYP46A1, can elicit all these multiple brain effects and become a potential therapeutic target for so many different brain disorders.

To begin to address this question, we recently proposed a mechanism (11, 33, 34), which herein we called the chain reaction hypothesis (Fig. 1). We suggested that since CYP46A1 controls brain cholesterol turnover (6, 9), CYP46A1 activity can alter at least three processes that we called “unifying“: *1*) sterol flux through the plasma membranes; *2*) acetyl-CoA production, and *3*) mevalonate production in the cholesterol biosynthesis pathway (Fig. 1). In turn, these unifying processes can affect other brain processes and represent secondary CYP46A1 effects (11, 33). We started to test our hypothesis and capitalized on our prior work, which identified low-dose anti-HIV drug efavirenz (EFV) as an allosteric activator of CYP46A1 in mouse and human brain (9, 16, 19, 23). This allowed us to use *Cyp46a1*^*−/−*^ (6) and EFV-treated 5XFAD mice (an Alzheimer’s disease model) (53) as animal models with lacking and increased, respectively, CYP46A1 activity, and hence altered brain cholesterol turnover and sterol flux (6, 19, 23). We obtained evidence that sterol flux and acetyl-CoA production depend, at least in part, on CYP46A1 activity which in turn affects other processes, namely, physicochemical properties of plasma membranes, protein phosphorylation, synaptic glutamate release, and acetylcholine biosynthesis (35, 36). In parallel, others showed that CYP46A1 activity can alter cholesterol content in lipid rafts (17, 37) and be important for some of the processes, which utilize the mevalonate pathway intermediates: long-term potentiation (LTP), higher order brain functions (17, 19, 22, 23, 29, 41, 42, 43, 44), motor functions (18, 21, 22), protein prenylation (40, 45), autophagy (20, 21), lysosomal function (21, 22, 44, 46), as well as malignant cell growth, and survival (25). Herein, we continued to test our hypothesis. We used 5XFAD mice, control and treated with low-dose EFV, and an unbiased multiomics approach to identify additional brain effects of CYP46A1 activation as indicated by changes in protein and metabolite abundance as well as protein acetylation. The data obtained expanded our knowledge about the CYP46A1 brain effects and provided further support for our chain reaction hypothesis.

**Fig. 1:**
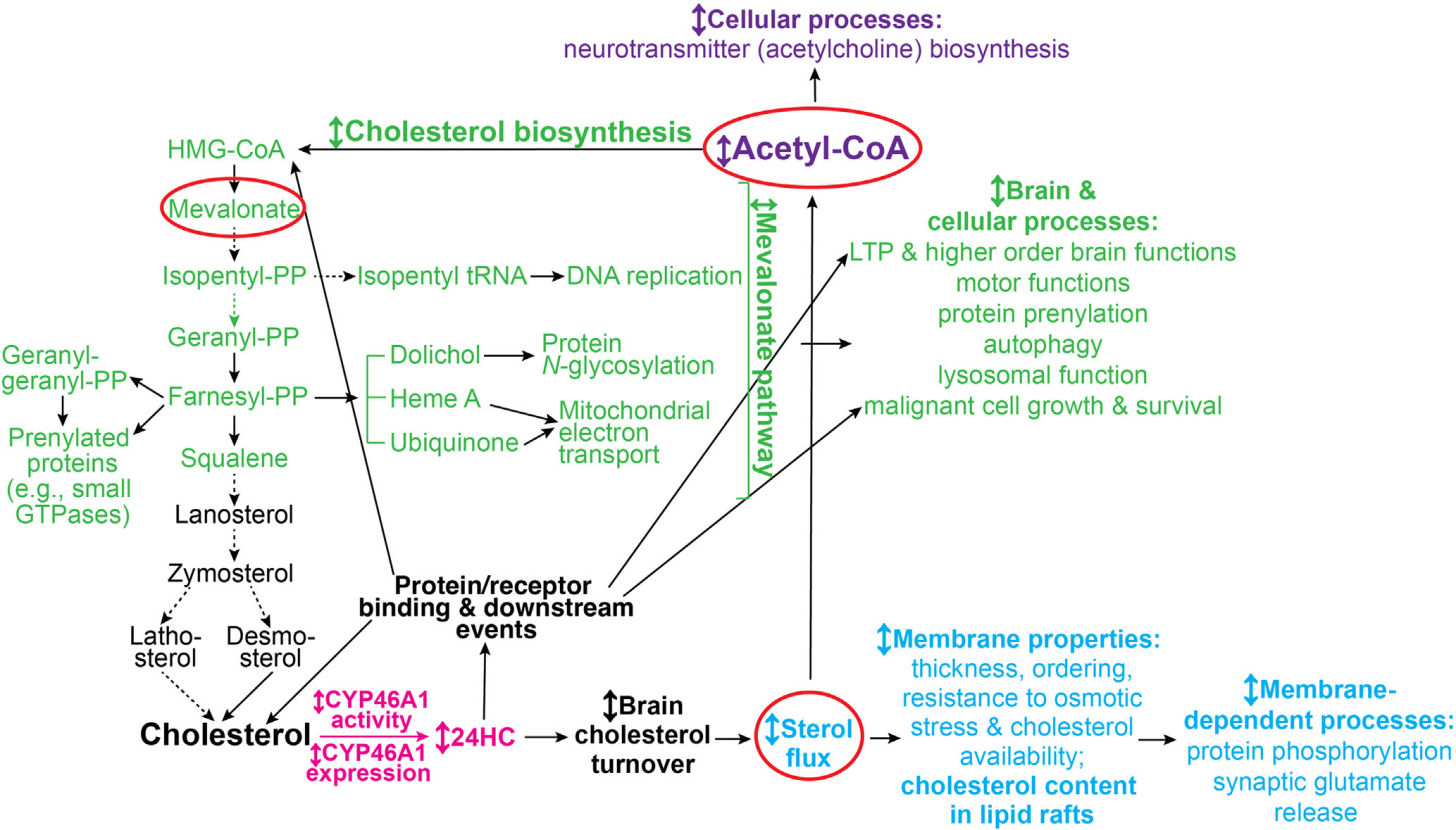
Schematic representation of the chain reaction hypothesis that explains a broad range of CYP46A1 targeting outcomes in the brain. The initial event, CYP46A1-mediated cholesterol 24-hydroxylation (in magenta), controls brain cholesterol turnover as it is tightly linked to cholesterol biosynthesis (6, 9). Brain cholesterol turnover may sequentially (or possibly in parallel) affect the three primary (unifying) processes (indicated by red ovals and colored), which are sterol flux through the plasma membranes (in cyan), acetyl-CoA production (in violet), and mevalonate production (in green) in the cholesterol biosynthesis pathway. These three primary processes can in turn affect other (secondary) biological processes (the same coloring as of the corresponding unifying process) as suggested by experimental evidence (reviewed in (11, 33)). Specifically, we already showed that in vivo, brain cholesterol turnover (and hence sterol flux in the brain) alters physicochemical properties of cellular membranes (membrane thickness, ordering, resistance to osmotic stress, and cholesterol availability) and several membrane-dependent events, including protein phosphorylation and synaptic glutamate release (35, 36). Others used cell cultures and demonstrated that *CYP46A1* overexpression can normalize cholesterol content in lipid rafts (17, 37). We then linked the brain sterol flux to acetyl-CoA production and identified acetylcholine biosynthesis as the secondary effect of the latter in the brain (36, 38). Finally, we linked acetyl-CoA production to cholesterol biosynthesis and mevalonate production as two acetyl-CoA molecules are necessary to synthesize mevalonate and then a third acetyl-CoA molecule is required for the next step, which is rate-limiting and irreversible (39). The mevalonate pathway yields nonsterol intermediates (geranylgeranyl pyrophosphate, farnesyl pyrophosphate, dolichol, heme A, and ubiquinone) that are essential for many brain processes (11, 40). Of them, several have already been shown to depend on CYP46A1 expression or activity: long-term potentiation and higher order brain functions (17, 19, 22, 23, 29, 41, 42, 43, 44), motor functions (18, 21, 22), protein prenylation (40, 45), autophagy (20, 21), lysosomal function (21, 22, 44, 46) plus malignant cell growth and survival (25). Notably, despite cholesterol supply from astrocytes (47), mevalonate synthesis is active in brain neurons to ensure the production of these essential compounds, and the brain neurons also express CYP46A1 (2) to provide in situ control of mevalonate synthesis and hence tight coupling of cholesterol production to its elimination (6, 41, 45). In addition to the three primary events, signaling via 24-hydroxycholesterol (24HC) could be important as well as represent a shunt pathway as 24HC is a biologically active molecule. Indeed, 24HC can bind to different proteins and receptors (e.g., insulin-induced gene 1 protein, liver X receptors, N-methyl-D-aspartate receptors, and G protein–coupled receptor 17) and thereby affect various downstream events such as cellular cholesterol biosynthesis, efflux, and transport, synaptic transmission, and maturation of oligodendrocyte progenitor cells (25, 48, 49, 50, 51, 52). Thus, altered CYP46A1 expression or activity in the brain can trigger a chain reaction, i.e., a sequence of primary events, which in turn elicit secondary effects and collectively underlie the multiplicity of the CYP46A1 brain effects. 24HC, 24-hydroxycholesterol; HMG-CoA, 3-hydroxy-3-methylglutaryl-CoA; LTP, long-term potentiation; PP, pyrophosphate. Dashed arrows indicate multiple steps; ↕, the up-down arrow indicates modulation (increase or decrease); Taken from (33) and modified. CYP46A1, cytochrome P450 46A1.

## Materials and methods

### Animals

5XFAD mice were on the B6SJL background (5XFAD^*Tg/0*^, stock No: 34840, the Jackson Laboratory, Bar Harbor, ME). These animals were hemizygous for the mutant (K670N, M671L, I716V, and V717I) human amyloid precursor protein 695 and mutant (M146L and L286V) human presenilin 1 (53). Male 5XFAD mice were crossed with B6SJL female mice (stock No: 100012, the Jackson Laboratory), after the B6SJL strain was bred out from the Pde6b^rd1^ mutation leading to early onset severe retinal degeneration and blindness (54). Only the F1 generation of hemizygous animals was used. EFV (the *S*-isomer, E425000, Toronto Research Chemical, Toronto, ON, Canada) was administered as described (23, 55), namely, in drinking water containing 0.0004% Tween 80 at a 0.1 mg/kg body weight/day dose. Mice were treated with EFV from 3 to 9 months of age as at 9 months, the Aβ production starts to plateau in this model (53). Control animals received aqueous 0.0004% Tween 80. Mice were maintained in a temperature- and humidity-controlled environment with 12 h light/12 h dark cycle in cages with water and food ad libitum. All animal experiments were approved by the Case Western Reserve University’s Institutional Animal Care and Use Committee and conformed to recommendations by the American Veterinary Association Panel on Euthanasia.

### Brain isolation and processing

Mice were fasted overnight, and the next morning euthanized. Mouse brains were isolated, rinsed in cold PBS, blotted, and processed as described (19, 56). Briefly, the brain stem beyond the cerebellum and olfactory bulb were removed, and the brain was dissected along the midline to obtain two hemispheres, which were flash-frozen in liquid nitrogen.

### Brain total and acetyl proteomes

All omics studies were carried out by Creative Proteomics (Shirley, NY), a proteomics MS company. Only male mice (five left brain hemispheres from five different mice per group) were used as no principal sex-based differences were previously found in the CYP46A1 activity effects on the brain (23, 35, 56), and our next similar study will be in female mice. Brain samples were processed as described previously for the retina (57). Briefly, left brain hemispheres were homogenized in 1 ml of the lysis buffer (8 M Urea, 50 mM Tris-HCl, pH 8.0, 50 mM NaCl, 1 mM DTT, and protease inhibitors) by beads grinder followed by tissue debris removal by centrifugation and the measurement of the protein concentration in the supernatant. Protein samples (2 mg of protein) were then diluted to 1.8 ml with the lysis buffer and reduced with 4.5 mM DTT at 37°C for 1 h. The solution was then diluted to 14.4 ml with 50 mM ammonium bicarbonate and subjected to an overnight digestion at 37°C with the MS-grade trypsin (Promega, Madison, WI, #VA9000) at an enzyme-to-substrate ratio of 1:200 (w/w). After digestion, trifluoroacetic acid was added to 1% final concentration, allowing precipitate to form at 4°C. The precipitate was then removed by centrifugation at 1,780 *g* for 15 min, and the peptides obtained were desalted on a C18 SPE column (Thermo Fisher Scientific, Inc. Waltham, MA). About 1% of each sample was used for the measurements of the relative protein abundance and the remaining 99% was used for acetyllysine enrichment.

For acetyllysine enrichment, peptide samples were dried by lyophilization and then resuspended in 50 mM Mops, pH 7.2, containing 50 mM NaCl and 10 mM Na_2_HPO_4_, Next, samples were incubated on a rotator for 4 h at 4°C with the anti-acetyllysine agarose beads (ImmuneChem Pharmaceuticals, Burnaby, Canada, ICP0388), which were prewashed four times in cold PBS. After the incubation, the beads were washed three times with cold 50 mM Mops, pH 7.2, containing 50 mM NaCl and 10 mM Na_2_HPO_4_, and then with HPLC-grade water (Sigma-Aldrich, Saint Louis, MO, #270733) four times. Peptides were eluted from the beads with 0.15% trifluoroacetic acid, lyophilized, and reconstituted in 0.1% (v/v) formic acid in LC-MS/MS grade water (Sigma-Aldrich, #1.15333). The peptide concentration was measured and diluted to a 0.1 μg/μl concentration across all samples.

The relative peptide or protein abundance was assessed by the label-free approach as described (57), Briefly, samples (1 μg of protein) were run on an Ultimate 3000 nano UHPLC system (Thermo Fisher Scientific, Inc.) coupled to a Q Exactive HF mass spectrometer (Thermo Fisher Scientific, Inc.). The MS was operated in the data-dependent acquisition mode. The full scan was performed between 300–1,650 m/z at the resolution of 60,000 at 200 m/z and the automatic gain control (AGC) target for the full scan at 3E^6^. The MS/MS scan was operated in Top 20 mode using the following settings: resolution 15,000 at 200 m/z; AGC target 1E5; maximum injection time 19 ms; normalized collision energy at 28%; isolation window of 1.4 Th; charge sate exclusion: unassigned, 1, >6; and dynamic exclusion 30 s.

Raw MS files were searched against the *Mus musculus* protein database (UniProt, https://www.uniprot.org/taxonomy/10090) using the MaxQuant software (Max Planck Institute of Biochemistry, Martinsried, Germany, version 2.2.0.0, https://www.maxquant.org). The maximum missed cleavage sites for trypsin was set to 5; Cys carbamidomethylation was set as a fixed modification; Met oxidation, protein N-terminal acetylation, and Lys acetylation were set as variable modifications. The precursor ion mass tolerance was set to 10 ppm, and the MS/MS tolerance was 0.5 Da.

For the brain total proteome, proteins were quantified and normalized using MaxLFQ as described (57). Proteins with a fold change of ≤0.83 and ≥1.2 or log_2_ fold change of ≤ −0.08 and ≥0.08 (an arbitrary cut off), and *P* values of ≤0.05 were considered as differentially expressed proteins (DEPs). Statistical significance was determined by one way ANOVA. For the brain acetylome, the false discovery rate was set to 1% for both peptide and protein levels, and the minimum required peptide length was set to seven amino acids. Match between runs feature was used to transfer identification. The intensity data were first logarithmized to obtain a normal or near-normal distribution. Student *t* test was then used to identify differentially acetylated peptides or proteins (DAPPs) with the *P* value of ≤0.05 and a fold change of ≤0.83 and ≥1.2 used as the threshold for significance.

### Untargeted brain metabolomics

Right brain hemispheres were mixed with 0.8 ml of 80% aqueous methanol and homogenized in a MM 400 Mixer Mill (Glen Mills Inc., Clifton, NJ) at 65 Hz for 90 s, followed by sonication on ice for 30 min. Samples were kept at −20°C for 1 h, then vortexed for 30 s, and subjected to centrifugation at 10,000 rpm, 4°C, for 10 min to remove precipitate. Supernatants (0.2 ml) were supplemented with 5 μl of 0.14 mg/ml 4-chloro-DL-phenylalanine (Sigma-Aldrich, # 7424-00-2) as internal standard to evaluate correct sample preparation and instrument variation. Samples were then filtered through a 0.22 μm filter and transferred to vials for ultra performance liquid chromatography (UPLC)-MS/MS analysis.

The analyte separation was performed on an Acquity UPLC I-Class system (Waters Corporation, Milford, MA) and an Acquity UPLC HSS T3 (100 × 2.1 mm × 1.8 μm) column coupled to a Q Exactive MS equipped with electrospray ionization. The mobile phase for UPLC was composed of solvent A (0.05% formic acid in water) and solvent B (100% acetonitrile). The chromatography gradient was run from 0% to 5% of solvent B over 1 min and then from 5% to 95% of solvent B over 11 min. Gradient elution was held in a 95% solvent B for the next 1.5 min and then was equilibrated in a 5% solvent B over 0.1 min, which washed the column for the next 2.4 min. The mobile phase flow rate was 0.3 ml/min at 40°C. Metabolomic analyses were performed in both positive and negative electrospray ionization (ESI) modes. The ESI positive mode was operated using following conditions: heater temperature of 300°C, sheath gas flow rate of 45 arb, aux gas flow rate of 15 arb, sweep gas flow rate of 1 arb, spray voltage of 3.0 KV, capillary temperature of 350°C, and S-Lens radio frequency level set constant at 30%. However, in the ESI negative mode, spray voltage and S-Lens radio frequency level were set at 3.2 KV and 60%, while other conditions remained same as the ESI positive mode. The settings for full scan data acquisition were as follows: resolution of 70,000 fwhm; AGC target of 3 × 10^6^; maximum injection time of 100 ms; scan range of m/z from 70 to 1,050 with polarity either negative or positive; and spectrum data type of centroid. Settings for data-dependent MS2 acquisition were as follows: resolution of 17,500 fwhm; AGC target of 1 × 10^5^; maximum injection time of 50 ms; isolation window of 1.7 m/z; loop count of 10; normalized collision energy of 15, 30, 45 eV; and the spectrum data type of centroid.

Raw UPLC/MS/MS data were processed with the Compound Discoverer 3.1 software (Thermo Fisher Scientific, https://www.thermofisher.com/us/en/industrial/mass-spectrometry/liquid-chromatography-mass-spectrometry-lc-ms/lc-ms-software/multiomics-data-analysis/compound-discoverer-software.html) that uses accurate mass data, isotope pattern matching, and mass library spectral searches for the structural annotation of small metabolites. The analysis of raw data included peak alignment, peak picking, and identification of each metabolite. For peak alignment, adaptive curve alignment model, allowable maximum shift of 2 min, and mass tolerance of 5 ppm were used. For peak picking, the minimum precursor mass was 0 Da and maximum precursor mass was 5,000 Da; the S/N threshold was 1.5; m/z range of 70–1,050 m/z; m/z width of 5 ppm; and a frame time width of 0.2 min. For peak identification, mass tolerance was 5 ppm; intensity tolerance was 30%; the S/N threshold was 3. Data were matched with the mzCloud (https://www.mzcloud.org) and mzVault (https://www.thermofisher.com/us/en/home/industrial/mass-spectrometry/liquid-chromatography-mass-spectrometry-lc-ms/lc-ms-software/mass-spectral-libraries.html) databases to obtain more accurate identification results. Thus, biological intelligence was used for compound annotation; therefore, compound identification is putative (58). Accordingly, the *sn*-position as well as the position and stereochemistry of double bonds are not indicated.

The positive and negative MS data were separately subjected to statistical analysis. Each metabolite abundance was expressed as a peak area, and the peak areas were normalized by the total peak area of each sample followed by a fold-change calculation. A two-tailed, unpaired Student’s *t* test was used to calculate the *P* value.

### Sterol quantifications

Cholesterol, 24HC, lathosterol, and desmosterol were quantified by isotope dilution gas chromatography-MS as described (59) using deuterated sterol analogs as internal standards.

### Multiomics data analyses

DEPs and DAPPs were analyzed for functional enrichment by the Panther (version 18, https://www.pantherdb.org) and STRING (version 12.0, https://string-db.org) software (60, 61). Integrated analysis of DEPs, DAPPs, and differentially abundant metabolites (DAMs) was carried out by the MetaboAnalyst (version 5, https://www.metaboanalyst.ca) software (62). Heatmaps and donut chart split representation were generated by the GraphPad Prism (version 10, https://www.graphpad.com/features) software.

### Statistics

Data from all available animals were used, and there was no exclusion of statistical outliers. The number of animals per group was always 5. Data were analyzed either by one way ANOVA or a two-tailed, unpaired Student’s *t* test. Statistical significance was defined as ∗*P* ≤0.05, ∗∗*P* ≤0.01, and ∗∗∗*P* ≤0.001.

## Results

### Differentially expressed proteins

**Fig. 2:**
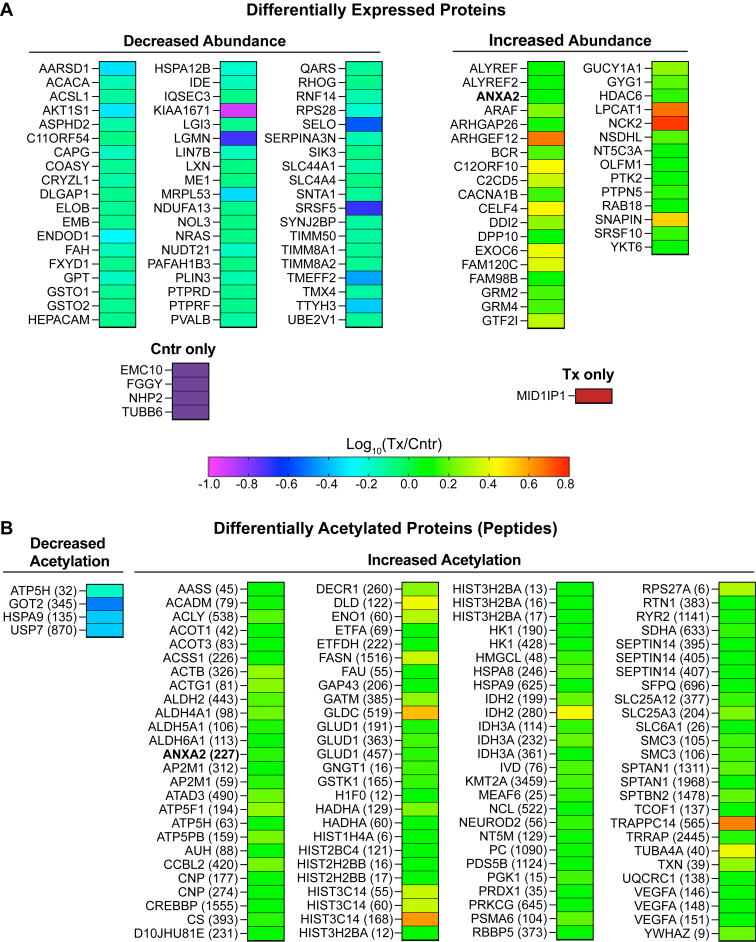
Changes in the brain proteome and acetylome in EFV-treated versus control 5XFAD mice. A: Differentially expressed proteins. B: Differentially acetylated proteins or peptides. The protein acetylation site is indicated in parenthesis. Samples from 5 male mice per genotype were used. Cntr, control; Tx, treated. EFV, efavirenz.

**Fig. 3:**
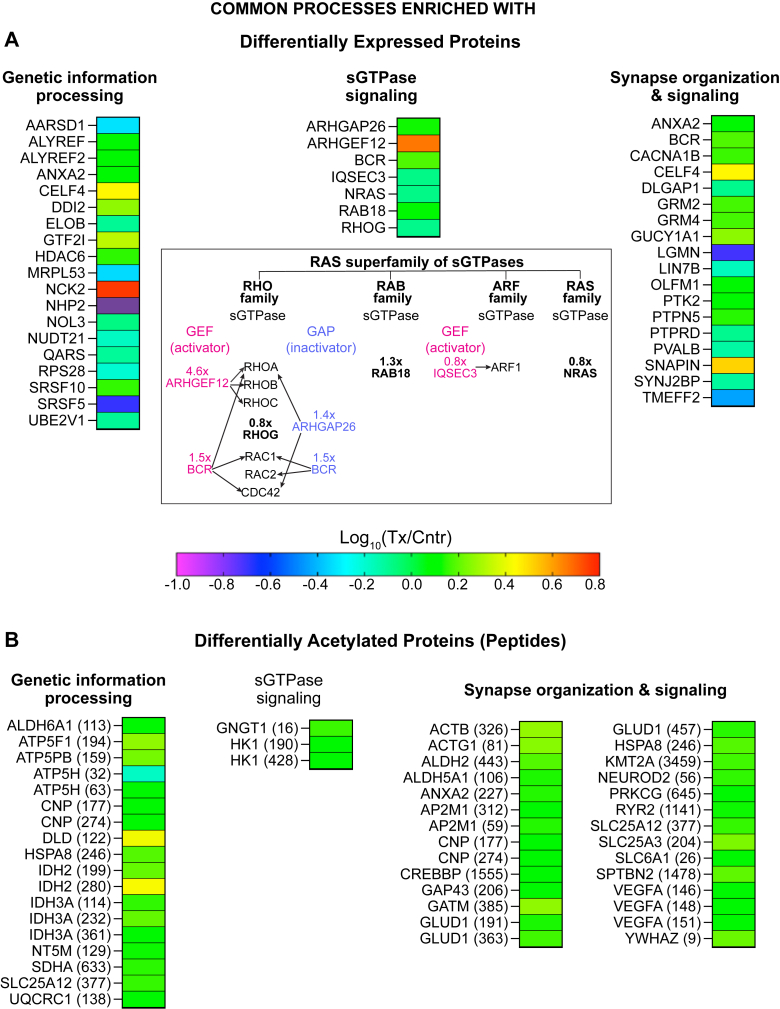
Common processes enriched with differentially expressed (A) and differentially acetylated proteins (B) in EFV-treated versus control 5XFAD mice. The protein acetylation site is indicated in parenthesis. Inset shows proteins with altered abundance that pertain to the RAS superfamily of small guanidine triphosphate binding protein (sGTPases). Arrows point to sGTPases that are regulated by guanine nucleotide exchange factors (GEF) and are inactivated by GTPase-activating proteins (GAPs) found as differentially expressed in the present work. BCR acts as both an activator and inactivator. Cntr, control; Tx, treated; x, a fold change in protein abundance in EFV-Tx versus Cntr 5XFAD mice. BCR, breakpoint cluster region; EFV, efavirenz; RAS, rat sarcoma.

**Fig. 4:**
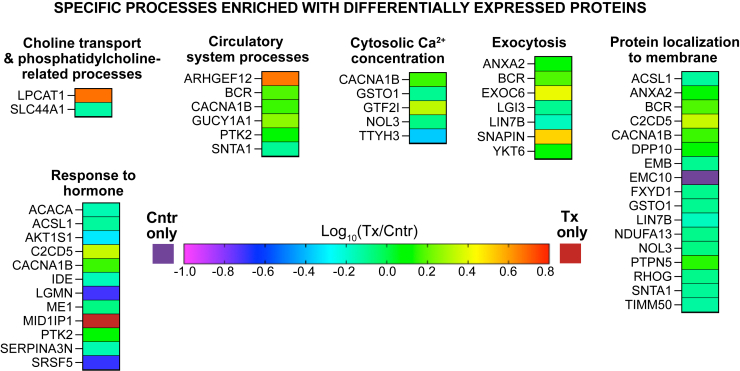
Processes enriched with differentially abundant proteins, which do not overlap with processes enriched with differentially acetylated proteins in EFV-treated versus control 5XFAD mice. Cntr, control; Tx, treated. EFV, efavirenz.

**Fig. 5:**
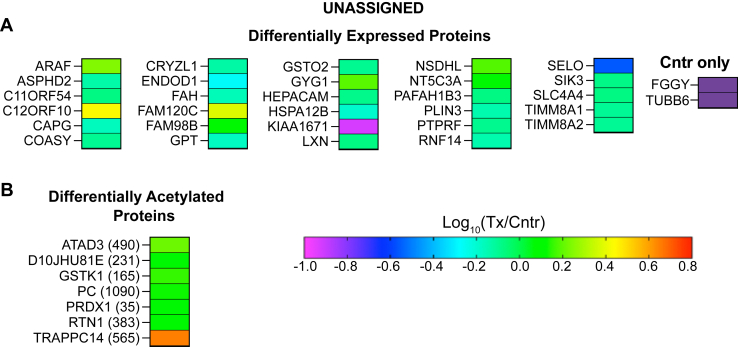
Differentially expressed (A) and differentially acetylated (B) proteins, which were not involved in functional enrichment in EFV-treated versus control 5XFAD mice. Cntr, control; Tx, treated. EFV, efavirenz.

About 3,130 proteins were found in the brain of 5XFAD mice (supplemental Table S1). Of them, 95 were differentially expressed in EFV-treated versus control mice: 57 had decreased abundance, 33 had increased abundance, 4 proteins were detected only in control mice, and one protein was detected only in EFV-treated mice. (Fig. 2A). These DEPs were then subjected to the functional enrichment analysis, which identified nine biological processes encompassing 63 DEPs. The identified processes were genetic information processing; small GTP-binding protein (sGTPase) signaling; synapse organization and signaling (Fig. 3A); choline transport and phosphatidylcholine (PC)-related processes; circulatory system processes; cytosolic Ca^2+^ concentration; exocytosis; protein localization to membrane; and response to hormone (Fig. 4). Notably, several of these processes were consistent with the CYP46A1 activity effects reported previously. These were genetic information transfer, which pertained to the previously shown CYP46A1 activity effects on gene expression (19, 20, 34, 56, 63); sGTPase signaling and protein localization to membrane, which pertained to the proposed CYP46A1 involvement in sGTPase prenylation (64); synapse organization and signaling, which pertained to the documented CYP46A1 activity effects on LTP, higher order brain functions (17, 19, 22, 23, 29, 41, 42, 43, 44), and motor functions (18, 21, 22); and choline transport, which pertained to the documented CYP46A1 activity effect on the levels of acetylcholine (38). Thus, the proteomics data were meaningful and suggested potential players mediating the CYP46A1 activity effects in the processes that were reported previously and processes that can potentially represent new CYP46A1 secondary effects. The significance of unassigned DEPs (Fig. 5) remains to be determined.

The DEP enrichment in sGTPase signaling provided unexpected insight as abundance of both sGTPases and regulators of their activity was affected by EFV treatment (Fig. 3A). sGTPases comprise the rat sarcoma (RAS) superfamily of G proteins, which include 5 families: RAS homolog (RHO); RAS-like proteins in brain (RAB); the ADP-ribosylation factor (ARF); RAS, and RAS-related nuclear protein (RAN) (65). The sGTPases are activated by guanine nucleotide exchange factors (GEFs) that promote their exchange of GDP for GTP and are inactivated by GTPase-activating proteins (GAPs) that enhance their intrinsic GTP hydrolysis (65). In addition, many sGTPases have to be prenylated with the nonsterol compounds produced in the mevalonate pathway (Fig. 1) for binding to membranes and activation of downstream effectors (65). The function of ten sGTPases from four families could be affected in EFV-treated versus control 5XFAD mice (Fig. 3A).

In the RHO family, the abundance of the sGTPase RAS-homology growth-related (RHOG) was modestly decreased (a 0.8-fold change). Conversely, the abundance of the several sGTPase regulators was increased. These were the GEF ARHGEF12 (RHO guanine nucleotide exchange factor 12 or leukemia associated RHO GEF), a 4.6-fold change, activates RHOA, RHOB, and RHOC (66); the GAP ARHGAP26 (RHO GTPase-activating protein 26 or GTPase regulator associated with focal adhesion kinase), a 1.4-fold change, inactivates RHOA and CDC42) (67); and a dual function breakpoint cluster region (BCR) protein, a 1.5-fold change. The BCR central domain activates RHOA, RAC1, and CDC42, whereas the BCR C terminus inactivates RAC1, RAC2, and CDC42) (68). In the RAB family, only the abundance of RAB18 was altered, namely increased 1.3-fold (Fig. 3A). Similarly, only one sGTPase could be affected in each, the ARF and RAS families (Fig. 3A). This could be due to decreased abundance of IQSEC3 (IQ motif and SEC7 domain-containing protein 3, a 0.8-fold change, the GEF for ARF1) and of the sGTPase neuroblastoma ras oncogene (NRAS, a 0.8-fold change). Future studies are necessary to ascertain the functional significance of altered abundance of sGTPases and their GEFs and GAPs found in the present work.

### Differentially acetylated peptides or proteins

**Fig. 6:**
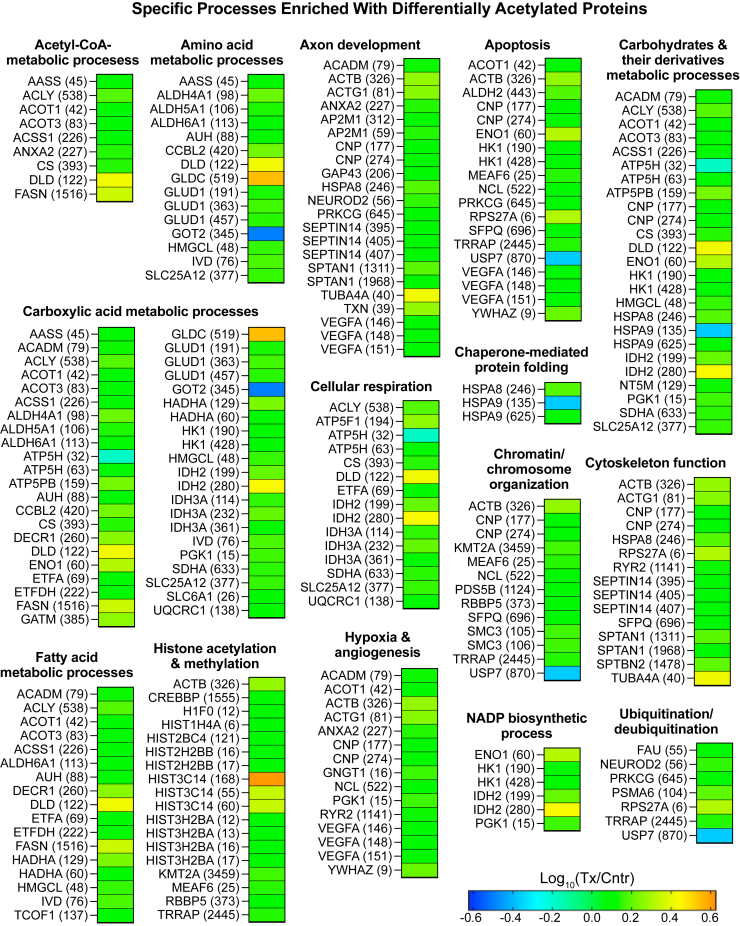
Biological processes enriched with differentially acetylated proteins, which do not overlap with processes enriched with differentially abundant proteins in EFV-treated versus control 5XFAD mice. Cntr, control; Tx, treated. EFV, efavirenz.

A total of 940 acetylated sites (peptides) were found in the brain of 5XFAD mice. Of them, 108 were differentially acetylated in EFV-treated versus control mice: 4 had decreased acetylation and 104 had increased acetylation (Fig. 2B). The differentially acetylated peptides were from 86 proteins, which did not overlap with DEPs, except ANXA2, which had both increased abundance (1.3-fold) and acetylation (1.4-fold) (Fig. 2). The analysis of DAPPs for functional enrichment identified 18 biological processes, which encompassed 79 proteins: 3 processes were common with those enriched with DEPs (Fig. 3B) and 15 were DAPP-specific (Fig. 6). The three common processes were genetic information processing; sGTPase signaling; and synapse organization and signaling. The 15 DAPP-specific processes were: acetyl-CoA-metabolic processes; amino acid metabolic processes; axon development; apoptosis; carbohydrates and their derivatives metabolic processes; carboxylic acid metabolic processes; cellular respiration; chaperone-mediated protein folding; chromatin/chromosome organization; cytoskeleton function; fatty acid metabolic processes; histone acetylation and methylation; hypoxia and angiogenesis; NADP biosynthetic process; and ubiquitination/deubiquitination.

The DAPP enrichment in histone acetylation was particularly informative as it encompassed acetylated histones. These were three of the four core histones: H2B (HIST2BC4, HIST2H2BB, and HIST3H2BA), H3 (HIST3C14), and H4 (HIST1H4A), and the linker histone H1 (H1F0) (Fig. 6). The affected residues were either in the histone tail domain (K12 in H1F0; K16 and K17 in HIST2H2BB; K12, K13, K16, and K17 in HIST3H2BA; and K6 in HIST1H4A) or in the histone globular domain (K121 in HIST2BC4 and K55, K60, and K168 in HIST3C14) (Fig. 6). Most of these acetylated sites were reported previously (69, 70). Plus, there was increased acetylation in the three important proteins that mediate histone acetylation: CREBBP (histone lysine acetyltransferase), MEAF6 (chromatin modification-related protein MEAF6), and TRRAP (transformation/transcription domain-associated protein) (Fig. 6); the latter two being the components of the NuA4 histone acetyltransferase complex (71). Thus, EFV treatment globally increased protein acetylation in the brain, including histone acetylation, an important secondary CYP46A1 activity effect. This effect agrees well with a documented increase in the brain acetyl-CoA levels in EFV-treated 5XFAD mice (36) and could be a potential mechanism for increased gene expression (19, 34) and protein abundance (Fig. 2) as a result of a CYP46A1 activation.

### Differentially abundant metabolites

**Fig. 7:**
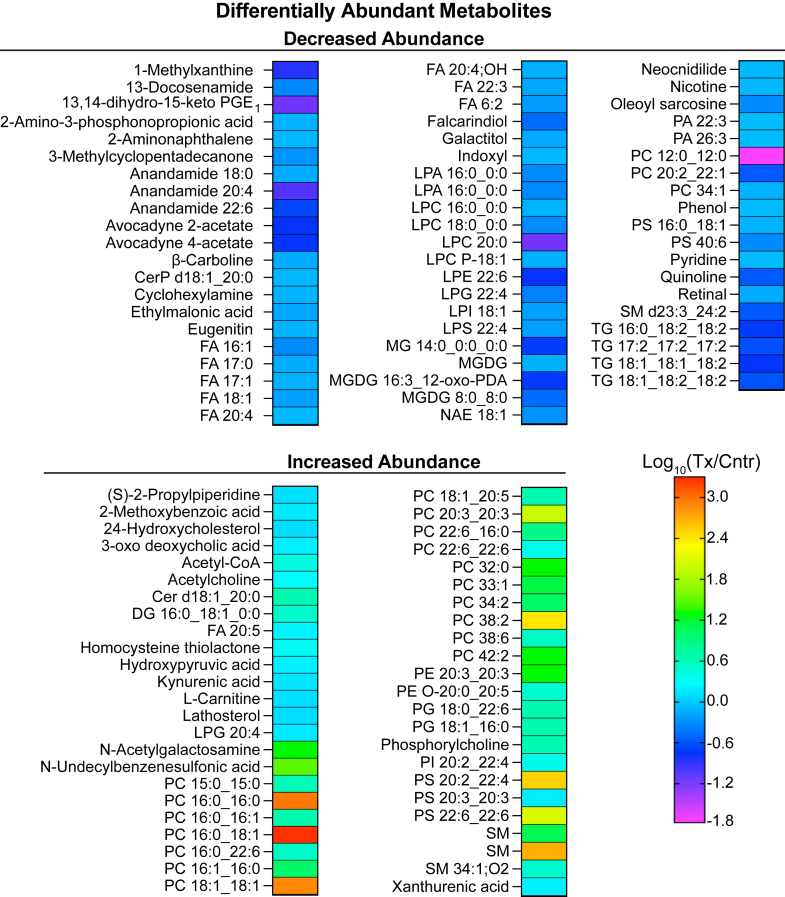
Changes in the brain metabolome in EFV-treated versus control 5XFAD mice. Samples from 5 male mice per genotype were used. 20-HETE, 20-hydroxyeicosatetraenoic acid; Cer, ceramide; CerP, ceramide 1-phosphate; Cntr, control; DG, diacylglycerol; EPA, eicosapentaenoic acid; FA, fatty acid; LPA, lysophosphatidic acid; LPC, lysophosphatidylcholine; LPE, lysophosphatidylethanolamine; LPI, lysophosphatidylinositol; LPL, lysoglycerophospholipid; LPS, lysophosphatidylserine; MG, monoacylglycerol; MGDG, monogalactosyldiacylglycerol; NAE, N-acyl ethanolamine; PA, phosphatidic acid; PC, phosphatidylcholine; PE, phosphatidylethanolamine; PG, phosphatidylglycerol; PGE_1_, prostaglandin E_1_; PI, phosphatidylinositol; PL, phospholipid; PS, phosphatidylserine; SM, sphingomyelin; TG, triacylglycerol; Tx, treated.

**Fig. 8:**
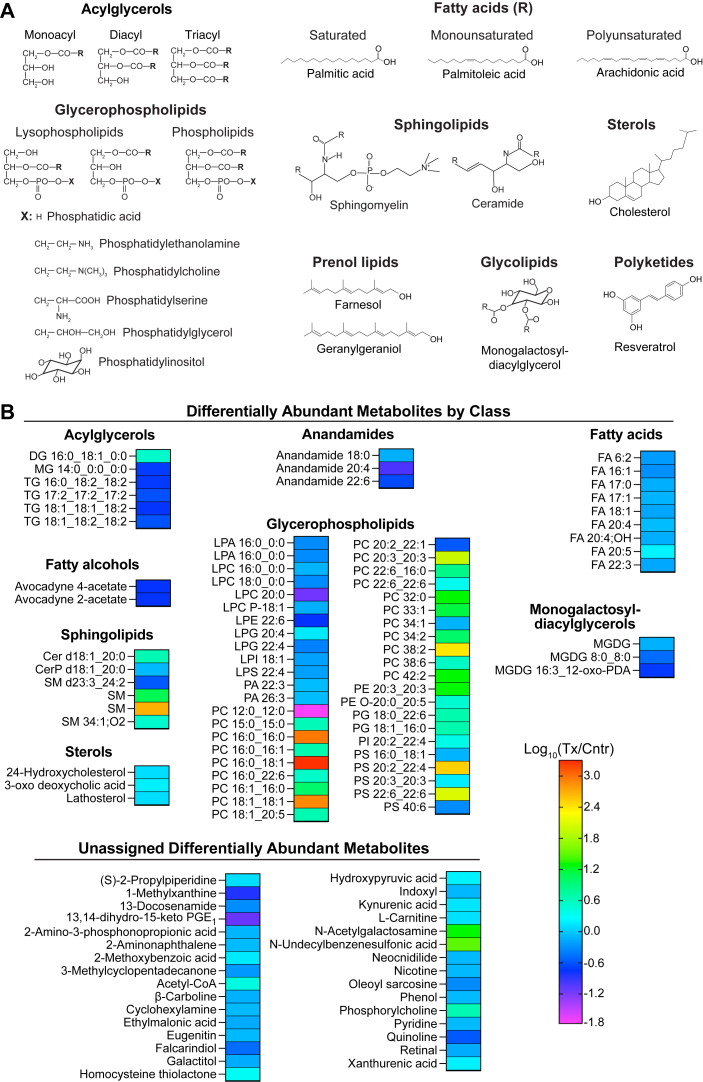
Major lipid classes (A) and the distribution of differentially abundant metabolites by lipid class in EFV-treated versus control 5XFAD mice (B). Representative compounds for each lipid class in A are also shown. The abbreviations are the same as in Fig. 7. EFV, efavirenz.

**Fig. 9:**
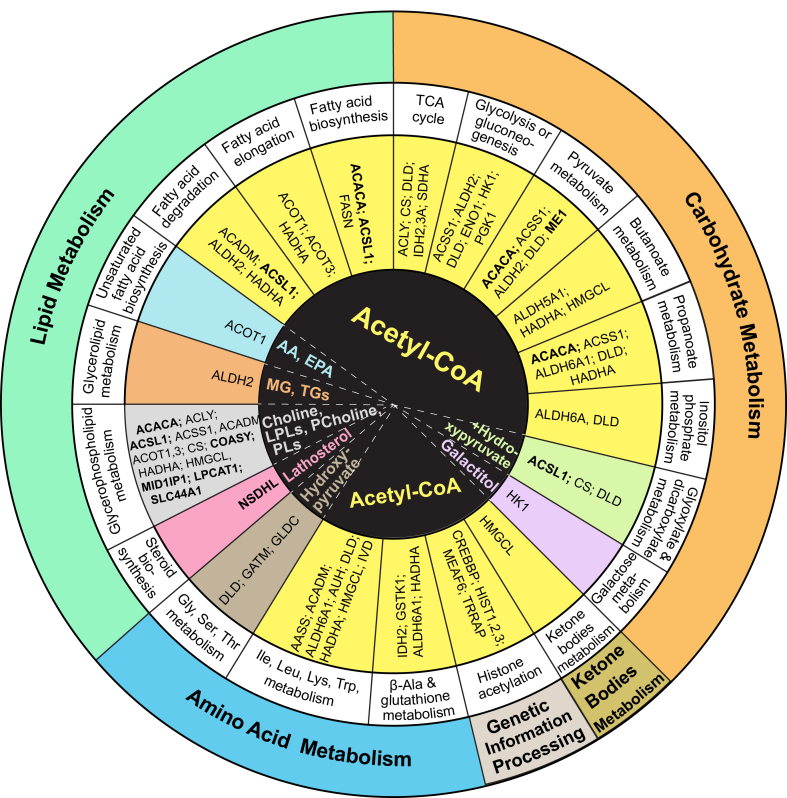
Integrated enrichment analysis. The donut chart split representation of functional enrichment based on combined analysis of differentially abundant metabolites (segments of the center black circle), differentially abundant proteins (in bold, segments of the inner ring), and differentially acetylated proteins (in regular font, segments of the inner ring). Specific processes and compound classes enriched with differentially abundant molecules from the integrated omics analysis are indicated in the segments of the middle and outer rings, respectively. PCholine, phosphorylcholine; the remaining abbreviations are as in Fig. 7.

A total of 6,119 compounds were found in the brain of 5XFAD mice. Of them, 108 were differentially abundant in EFV-treated versus control mice: 61 had decreased abundance and 47 had increased abundance (Fig. 7). The identified DAMs were mostly lipids (∼70% of the DAMs) and only their certain classes (Fig. 8A). About 43% of the DAMs were glycerophospholipids, namely lysoglycerophospholipids (LPLs) and phospholipids (PLs). The affected LPLs were lysophosphatidic acid, lysophosphatidylcholine, lysophosphatidylethanolamine, lysophosphatidylglycerol, lysophosphatidylinositol, and lysophosphatidylserine. The affected PLs were phosphatidic acid, PC, phosphatidylethanolamine, phosphatidylglycerol, phosphatidylinositol, and phosphatidylserine (Fig. 8B). Fatty acids, acylglycerols, and sphingolipids represented 9.1%, 5.5%, and 5.5%, respectively, of the DAMs, whereas anandamide (fatty acid derivatives), sterols, and fatty alcohols each accounted for less than 2% of DAMs (Fig. 8B). The remaining 33 DAMs (∼30%) were from other classes of organic compounds. DAMs were analyzed for functional enrichment along with DEPs and DAPPs. This integrated pathway analysis identified 20 biological processes, of which eight pertained to carbohydrate metabolism, seven pertained to lipid metabolism, three pertained to amino acid metabolism, and one pertained to each genetic information processing and ketone body metabolism (Fig. 9). The specific processes affected within the carbohydrate metabolism were the tricarboxylic acid cycle, glycolysis or gluconeogenesis, as well as metabolism of pyruvate, butanoate, propanoate, inositol phosphate, glyoxylate, dicarboxylate, and galactose (Fig. 9). The processes affected within the lipid metabolism were biosynthesis of steroids, fatty acids, and unsaturated fatty acids, metabolism of glycerophospholipids and glycerolipids, as well as fatty acid elongation, and degradation (Fig. 9). Finally, metabolism of the three groups of amino acids was affected within the amino acid metabolism: *1*) Gly, Ser, and Thr; *2*) Ile, Leu, Lys, and Trp; and *3*) β-Ala and glutathione (Fig. 9).

Notably, of the 20 enriched biological processes, 14 (histone acetylation plus 13 additional processes) pertained to acetyl-CoA, and the remaining 7 were based on altered abundance of other metabolites (galactitol, hydroxypyruvate, lathosterol, PL-related compounds, acylglycerols, and fatty acids, Fig. 9). Of these remaining seven processes, glycerophospholipid metabolism encompassed the highest number of DAMs (56) followed by glycerolipid metabolism, encompassing 6 DAMs (monoacylglycerols, diacylglycerols, or triacylglycerols, Fig. 8B). Thus, the integrated metabolomics, proteomics, and acetylomics approach identified endogenous compounds in EFV-treated versus control 5XFAD mice with altered levels and the biological processes and proteins that could be associated with these DAMs. Most of the identified biological processes (14 of 20) related either to acetyl-CoA production or utilization, and likely represented the secondary CYP46A1 activity effects.

## Discussion

Herein, we continued to focus on the CYP46A1 activity effects and used the same set of brain samples to simultaneously study changes in the tissue proteome, acetylproteome, and metabolome in EFV-treated versus control 5XFAD mice. We obtained unbiased mechanistic insights into specific processes and molecules that could be affected by a CYP46A1 activity increase, and thereby further detailed and supported our chain reaction hypothesis (Fig. 1). We found that many processes (mainly metabolism of lipids, carbohydrates, amino acids, and ketone bodies, Fig. 9) pertain to acetyl-CoA homeostasis and even more processes could depend on abundance or acetylation of specific proteins (Fig. 3, Fig. 4 and 6). Only the most informative processes and molecules that were identified by our multiomics approach will be discussed below.

**Fig. 10:**
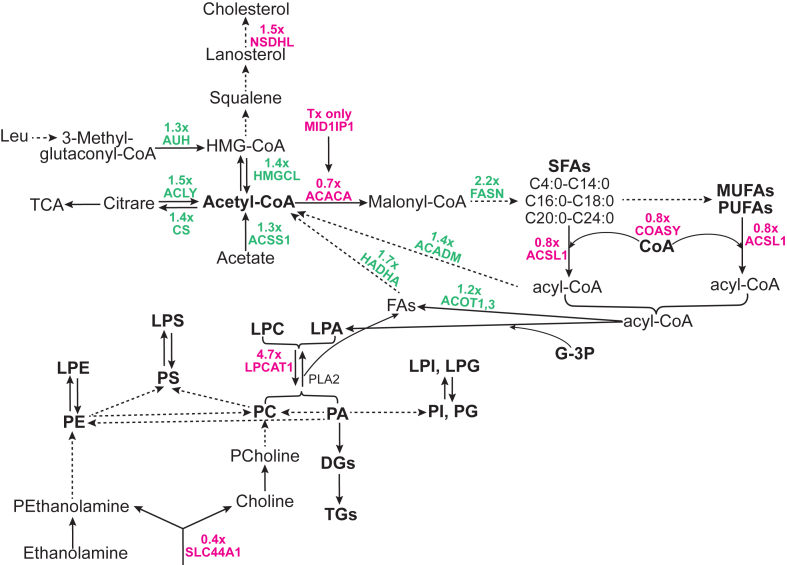
Schematic representation of the acetyl-CoA involvement in sterol, fatty acid, acylglycerol, and phospholipid metabolism. Some of the enzymatic reactions catalyzed by differentially abundant (in magenta) and differentially acetylated (in green) proteins that lead to production or utilization of acetyl-CoA. Dashed arrows indicate multiple steps. acetyl-CoA carboxylase 1, MID1IP1, acyl-CoA synthetase long chain family member 1, COASY, LPCAT1, and SCL44A1 are described in the main text. The preferred substrates for LPCAT1 in vitro are medium to long chain saturated LPCs (C12:0-C18:0) and the unsaturated LPC C18:1. The preferred acyl donors for LPCAT1 are long chain saturated fatty acyl-CoAs (C6:0-C16:0) and the unsaturated C18-, C20-, and C22-containing CoAs (C18:1, C18:2, C18:3, C20:4, and C22:6, respectively) (72). ACADM (mitochondrial medium-chain specific acyl-CoA dehydrogenase) catalyzes the first step of mitochondrial fatty acid β-oxidation. ACLY (ATP-citrate synthase) catalyzes the cleavage of citrate into oxaloacetate and acetyl-CoA, thus connecting glucose metabolism with lipid synthesis. ACOT1,3 (acyl-coenzyme A thioesterase 1,3) catalyzes the hydrolysis of acyl-CoAs into free fatty acids and CoA; more active toward saturated and unsaturated long chain fatty acyl-CoAs. ACSS1 (mitochondrial acetyl-coenzyme A synthetase 2-like) catalyzes the synthesis of acetyl-CoA from short-chain fatty acids and uses acetate as preferred substrate. AUH (mitochondrial methylglutaconyl-CoA hydratase) catalyzes the fifth step in the Leu degradation pathway. CS (mitochondrial citrate synthase) catalyzes the reaction reverse to that of ACLY. FASN (fatty acid synthase) is the critical enzyme responsible for de novo fatty acid synthesis. HADHA is the α subunit of the mitochondrial trifunctional enzyme that catalyzes the last three of the four reactions in the mitochondrial fatty acid β-oxidation. HMGCL (mitochondrial 3-hydroxymethyl-3-methylglutaryl-CoA lyase) catalyzes the cleavage of 3-hydroxy-3-methylglutaryl-CoA into acetyl-CoA and acetoacetate, a key step in ketone bodies production and a terminal step in Leu catabolism. NSDHL (sterol-4-α-carboxylate 3-dehydrogenase) catalyzes oxidative decarboxylation of the sterol C4 methyl groups in the postsqualene portion of cholesterol biosynthesis. G3P, glycerol-3-phosphate; HMG-CoA, 3-hydroxy-3-methylglutaryl coenzyme A; DAG, diacylglycerol; FA, free fatty acid; PCholine, phosphoryl choline; PE, phosphatidylethanolamine; PEthanolamine, phosphoryl ethanolamine; PLA2, phospholipase 2; PUFA, polyunsaturated fatty acids; SFA, saturated fatty acids, MUFA, monounsaturated fatty acids; TAG, triacylglycerol; TCA, the tricarboxylic acid cycle; x, a fold change in abundance or acetylation in EFV-treated versus control 5XFAD mice; the remaining abbreviations are as in Fig. 7. EFV, efavirenz; LPCAT1, lysophosphatidylcholine acyltransferase 1; MID1IP1, mid1-interacting protein 1.

Perhaps, the most unambiguous effect of CYP46A1 activity, secondary to acetyl-CoA production, was observed on the biosynthesis of different PLs from their corresponding LPLs. This effect was consistent with an overall decrease and increase in abundance of LPLs and PLs, respectively (Fig. 8B); the ability of different PL classes to undergo interconversions (Fig. 10) (73); a 4.7-fold increase in abundance of lysophosphatidylcholine acyltransferase 1 (LPCAT1) (Fig. 2); and substrate preference of this enzyme (72, 73, 74, 75). Indeed, among all the DAMs, the highest increases in EFV-treated versus control mice were in abundance of PLs and particularly the PC species 16:0_16:0 (795-fold), 16:0_18:1 (1,616-fold), and 18:1_18:1 (673-fold) (Fig. 8B) that are produced from the preferred substrates and acyl-CoA donors of LPCAT1 (see Fig. 10 legend). Of the nonglycerophospholipids, the highest increase in abundance was in one of the sphingomyelins (438-fold) (Fig. 8B), possibly a reflection that the phosphorylcholine group of PC is required for the biosynthesis of sphingomyelin from ceramide (76).

Besides LPCAT1, EFV-treated versus control mice had an altered abundance of several other important enzymes involved in the PL production either from choline (SLC44A1) or LPL acetyl-CoA carboxylase 1 (ACACA), mid1-interacting protein 1 (MID1IP1), acyl-CoA synthetase long chain family member 1 (ACSL1), and coenzyme A synthase (COASY)) (Figs. 2A and 10). SLC44A1 (choline transporter-like protein 1) transports choline and ethanolamine inside the cells and mitochondria (77, 78, 79) to initiate the main (Kennedy) pathway of PC biosynthesis in eukaryotes (80). The SLC44A1 abundance was decreased 0.4-fold (Fig. 2A); nevertheless, the content of phosphorylcholine, which is produced from choline, was increased 4.0-fold (Figs. 8B and 10). Further, ACACA, MID1IP1, ACSL1, and COASY are all involved in the biosynthesis of acyl-CoAs necessary for the PL biosynthesis from LPL (Fig. 10). ACACA converts acetyl-CoA to malonyl-CoA, the first and rate-limiting step in de novo fatty acid biosynthesis (Fig. 10). The ACACA abundance was decreased 0.7-fold (Fig. 2A). Yet this decrease was perhaps compensated by increased expression of MID1IP1, which was detected only in EFV-treated mice (Fig. 2A) and which posttranslationally upregulates ACACA activity and de novo fatty acid synthesis in vivo (81). ACSL1 (long chain fatty acid CoA ligase 1) preferentially activates the C16:0, C18:0, C18:1, C18:2, and C20:4 fatty acids (82) and utilizes CoA, which is synthesized in the pathway, where COASY (bifunctional coenzyme A synthase) catalyzes the final steps (83) (Fig. 10). EFV treatment reduced the ACSL1 and COASY abundance only modestly (each 0.8-fold, Fig. 2A) and did not lead to an accumulation, but rather a decrease, in the brain free fatty acid content (Fig. 8B). This was possibly because of the fatty acid utilization by other ACSL isoforms that act on the substrates overlapping with those of ACSL1 (82, 84, 85). The only exception was eicosapentaenoic acid (a 1.6-fold increase in abundance), which is obtained from the diet either directly or from α-linolenic acid (C18:3); the latter being a substate in eukaryotes for the endogenous eicosapentaenoic acid biosynthesis (86). Thus, besides LPCAT1, only increased abundance of MID1IP1 in EFV-treated versus control 5XFAD mice could facilitate their production of acyl-CoAs from acetyl-CoA and thereby increase the conversion of LPLs into PLs. Of course, of importance could be the increased acetylation of various proteins involved in acetyl-CoA and fatty acid homeostasis (Figs. 2B and 10). Yet, the consequences of these acetylations are less clear and cannot be unambiguously interpreted (87) as discussed later.

Previous studies showed that the brain (hippocampal) lipidome was affected in WT mice, in which 70% of *Cyp46a1* expression was reduced after the hippocampal injection of an adenoviral plasmid containing the short-hairpin RNA against *Cyp46a1* (44, 46). The treated animals had increased hippocampal abundance of phosphatidylethanolamines, PCs, and sphingolipids (including sulfatides, ceramides, glucosylceramides, and gangliosides GM1), as well as of diacylglycerols with long and unsaturated fatty acyls (46). Also, there were increases in the mRNA levels of some of the enzymes involved in the PC (*Pcyt1a* and *Pcyt1b*) and sphingolipid (*Lass2*, *Smpd1*, and *Smpd3*) metabolism (46). The observed changes were linked to cholesterol accumulation in hippocampal neurons, and therefore these major dysregulations of lipid homeostasis. Our study suggests that perhaps not only cholesterol accumulation, but CYP46A1 activity per se could contribute, at least in part, to the observed changes in the hippocampal lipidome, some of which overlap with those in EFV-treated mice, and some are genotype (context)-specific.

The brain lipidome is known to be altered in AD and mouse models of this disease and therefore could be involved in AD pathogenesis by affecting the membrane lipid content and hence membrane-bound proteins involved in the amyloid β production. In addition, membrane lipids can affect the amyloid β propensity to aggregate and in turn be reciprocally affected by the amyloid β peptides (88, 89, 90). Yet, it is quite difficult to compare different studies of brain lipidome because of the variability in age, sex, disease stage, animal model, analytical technique, as well as brain regions in human, or mouse samples that were analyzed (90). Nevertheless, despite all this variability, some general trends in human and mouse brain samples with AD manifestations were revealed: an increase in LPLs, sphingomyelins, gangliosides, and monounsaturated fatty acids and a decrease in sulfatides (90). This knowledge raises a question whether a decrease in the brain LPL abundance and an increase in the PL abundance (the most unambiguous EFV effect in 5XFAD mice) a beneficial or detrimental pharmacologic outcome? We suggest that this outcome is beneficial as increased LPL levels were shown to promote the formation of amyloid β plaques and neurofibrillary tangles, plus neuroinflammation, demyelination, motor function defects, and vascular barrier disruption (91, 92). Moreover, 9-month old 5XFAD mice were found to have an increase in the brain LPL species and a decrease in the corresponding PC species due to the phospholipase 2 activation (93). Yet, EFV treatment elicited an opposite effect and involved a different enzyme (LPCAT1). As LPCAT1 and phospholipase 2 catalyze opposite reactions, namely glycerophospholipids acylation and deacylation (Fig. 10), respectively, increased abundance of LPCAT1 likely diminished aberrant LPL production in 5XFAD mice, increased the diversity of the glycerophospholipid acyl chains, and facilitated glycerophospholipid remodeling (94, 95), apparently all beneficial pharmacologic outcomes.

Increased protein acetylation (the acetyl group transfer from acetyl-CoA to the protein Lys residue) was another unambiguous CYP46A1 activity effect, observed in 95% of proteins with altered acetylation in EFV-treated versus control 5XFAD mice (Fig. 2B). Acetylation is one of the most common posttranslational modifications of proteins (96, 97), and we found many processes enriched with DAPPs after EFV treatment (Figs. 3 and 6). These processes included production and utilization of acetyl-CoA and fatty acids (Fig. 10), glycolysis, the tricarboxylic acid cycle, amino acid catabolism, and many other (Fig. 6). However, when multiple proteins in a pathway have altered acetylation, and the acetylation of multiple Lys residues within the same protein is affected, the net effect of acetylation on protein function and pathway is usually difficult to predict (87). An exception is histone acetylation, which weakens the interaction between DNA and histones and generally increases gene transcription (69, 70). Histone acetylation is a part of such important processes in the brain as memory formation, consolidation, and synaptic plasticity; yet it is decreased in both aging and AD (98, 99). Of the four core histones (H2A, H2B, H3, and H4), variants of the three core histones (H2B, H3, and H4) and of the linker histone H1 had increased acetylation as a result of EFV treatment. Hence, further studies are necessary to ascertain whether increased protein acetylation found in the present work contributes to memory improvements and other processes in EFV-treated versus control 5XFAD mice (19, 23).

Of the nonhistones, tubulin α-4A chain was the first acetylated protein identified (69). The effect of its increased acetylation at K40 (2.6-fold, Fig. 2B) is known, increased protein stability, which improves axonal transport, usually impaired in neurodegenerative disorders (100). Tubulin α-4A chain is deacetylated by HDAC6 (101) as are the Lys residues in the tail domain of the core histones (102). The HDAC6 abundance was increased 1.4-fold in EFV-treated versus control 5XFAD mice but was not altered in 5XFAD versus WT mice (103). Hence increased HDAC6 abundance in EFV-treated versus control 5XFAD mice could be a compensatory response to increased acetylation of the HDAC6 substrates.

Finally, it is worth discussing the EFV treatment effect on sGTPases (Fig. 3A). Previously, CYP46A1 overexpression in cell culture and mouse brain was shown to increase neuronal isoprenoid synthesis (Fig. 1) and hence prenylation and membrane binding of several sGTPases (RHOA, RAC1, CDC42, and RAB8) (64). In this work, EFV treatment altered abundance of ether sGTPases (RHOG, RAB18, and NRAS) or their activators and inactivators (ARHGEF12, ARHGAP26, BCR, and IQSEC3) (Fig. 3A). In the RHO family, RHOG participates in cytoskeletal reorganization in various cell types and thereby regulates neurite outgrowth, cell survival, and migration (e.g., glioblastoma cell invasion) (104, 105). ARHGEF12 plays critical roles in the cyclic-stretch-induced cell and stress fiber reorientation responses, mesenchymal stem cell fate, cell migration, and invasion (106). ARHGAP26 attenuates cellular response to integrin-extracellular interactions and plays a role in lipid droplet biogenesis. Importantly, the ARHGAP26 single nucleotide polymorphisms were reported to be associated with AD, Parkinson’s, neuropsychiatric, and cardiovascular diseases (67, 107, 108). Finally, BCR is involved in the LTP maintenance, learning, and memory (109). BCR resides at excitatory synapses and interacts with postsynaptic density protein 95 (PSD-95), an abundant postsynaptic scaffold protein (109), whose levels were increased in EFV-treated versus control mice (34). In the RAB family, RAB18 has been implicated in a wide variety of processes including autophagy, granule secretion, lipid droplet biogenesis, and sterol mobilization and biosynthesis (110, 111, 112). In the ARF family, IQSEC3 regulates the formation of vesicle coats at different steps in exocytosis and endocytosis (113). In the RAS family, mutations in NRAS play critical roles in human oncogenesis (113, 114). Thus, similar to CYP46A1 overexpression (64), sGTPase signaling could be affected by EFV treatment. However, it needs to be clarified whether the observed changes in protein abundance is a consequence of increased prenylation and membrane binding of sGTPases or a totally different CYP46A1 activity effect on sGTPases.

With respect to other biological process, which were enriched with differentially abundant molecules in EFV-treated versus control mice, their complexity does not always allow unambiguous interpretation of the omics data, even when an integrated multiomics approach was used. Yet, there is a growing interest in CYP46A1, and we set up a framework for systematic mechanistic studies of this fascinating enzyme by proposing the chain reaction hypothesis (Fig. 1). Data from different laboratories will keep accumulating, and this knowledge will further detail our hypothesis or even suggest additional unifying mechanisms. This laboratory will continue to test the chain reaction hypothesis and will apply the multiomic approach to studies of the brain of different groups of mice to identify conditional and unconditional CYP46A1 activity effects (*e.g.*, female 5XFAD mice (control and EFV-treated); female and male B6SJL mice (control and EFV-treated), a background strain for 5XFAD mice; and both sexes of *Cyp46a1*^*−/−*^ mice versus WT). In addition, we will investigate the brain prenylome in different groups of animals and analyze specific brain regions in some of the groups.

In summary, we previously proposed the chain reaction hypothesis to explain the multiplicity of the brain effects of CYP46A1 activity modulation. Herein, we continued to test this hypothesis and investigated changes in the brain proteome, acetylome, and metabolome of 5XFAD mice treated with the CYP46A1 activator EFV. We identified DEPs, DAPP, and DAMs in EFV-treated versus control 5XFAD mice, and many biological processes enriched with these differentially abundant molecules. We suggest that PL metabolism and histone acetylation are important CYP46A1 activity effects secondary to increased acetyl-CoA abundance, and that these effects likely represent the positive EFV treatment outcomes. The additional secondary CYP46A1 activity effects and proteins mediating these effects were suggested as well and need to be further studied for functional significance and the association with the primary CYP46A1 activity effects. Overall, CYP46A1 was confirmed to be a key protein in the brain and a promising therapeutic target for at least AD.

## Data availability

The raw proteomics data that support the findings of this study will be shared upon request.

## Supplemental data

This article contains supplemental data.

## Conflict of interest

The other authors declare that they have no conflicts of interest with the contents of this article.
